# Mechanisms by which chloropicrin fumigation promotes soil potassium conversion and absorption

**DOI:** 10.3389/fmicb.2023.1208973

**Published:** 2023-07-13

**Authors:** Yang Sun, Rong Zeng, Wensheng Fang, Jvling Hua, Shuijin Huang, Qiuxia Wang, Aocheng Cao, Feng Zhu, Haiyan Zhang

**Affiliations:** ^1^Institute of Plant Protection, Jiangxi Academy of Agricultural Sciences, Nanchang, China; ^2^Institute of Plant Protection, Chinese Academy of Agricultural Sciences, Beijing, China; ^3^Institute of Plant Protection, Guizhou Academy of Agricultural Sciences, Guiyang, China

**Keywords:** soil fumigation, soil potassium, soil microorganism, chloropicrin, tomato

## Abstract

Fumigation of soil using chloropicrin has been proven to significantly affect soil nutrient cycling, but the mechanism by which soil potassium conversion and plant uptake is promoted remains unclear. In this study, we conducted a fumigation experiment to investigate the effects of chloropicrin soil fumigation on the conversion of soil potassium post-fumigation (days 7–70), and its mechanisms, tomatos were planted in fumigated and non-fumigated soils to enable further comparisons. Results showed that the content of rapidly available potassium and available potassium decreased by 16–24% and 17–23% at day 28 respectively, when tomato was planted in chloropicrin-fumigated soils compared to the non-fumigated soils. The potassium content of tomato planted in fumigated soil was significantly higher than that planted in non-fumigated soil (30.3 vs. 21.9 mg g^−1^ dry weight). Chloropicrin fumigation resulted in a significant change in the soil bacterial and fungal community structures, and trigged a long-term (at least 70-day) decrease in microbial diversity. Network analysis showed that chloropicrin soil fumigation changed microbial co-occurrence patterns by decreasing bacterial total links, nodes, and average degree, and increasing fungal total links, nodes, and average degree. Chloropicrin fumigation caused significant changes in the relative abundance of *Bacillus* species, which are involved in potassium dissolution. Structural equation model (SEM) suggested that fumigation with chloropicrin enhanced the contribution of soil potassium to tomato growth and reduced the contribution of bacterial communities. Together, the results of our study help in understanding the crop yield enhancement mechanism of soil fumigation.

## 1. Introduction

Chloropicrin is a soil fumigant commonly used in agriculture for controlling pests, pathogens, and weeds. It is often used to conditions soil before planting crops, to create a clean environment for seed germination and plant growth (Cao et al., 2011). Chloropicrin soil fumigation not only effectively controls the occurrence of soilborne diseases but also significantly increases crop yield (Cao et al., 2022; Fang et al., 2023; Wang et al., 2023). By affecting the abundance and activity of microorganisms, soil fumigation affects soil nutrient use, including the C, N, and P cycles, for example changing the form and content of soil-available nitrogen and phosphorus (Fang et al., 2018a,b,c, 2019; Huang et al., 2019, 2020). However, the effect of chloropicrin fumigation on soil potassium conversion and its mechanisms are unknown.

Soil potassium is one of the essential macronutrients required for plant growth and development. It plays a crucial role in various physiological processes within plants including providing nutrition for plant growth, maintaining proper osmotic potential, enhancing plant resistance to diseases and pests, extending the shelf-life of harvested crops (Mengel and Busch, 1982; Martin and Sparks, 1985; Sparks and Huang, 1985; Marschner, 2011; Prajapati and Modi, 2012; Saha et al., 2016; Xu et al., 2020). Notably, different plant species have varying potassium requirements, and soil conditions can affect the availability of potassium to plants. Soil is an important source of potassium for plants, with an average potassium content of 2.6%, making it one of the most abundant macronutrients (Dang et al., 2014). Potassium exists in various forms in soil, but mainly in inorganic forms. According to its availability to crops, it is divided into three types: rapidly available potassium (including water-soluble potassium and exchangeable potassium); slowly available potassium; and relatively ineffective potassium (mineral structure potassium) (Jin, 1993; Bao, 2018). These types of soil potassium can be transformed into each other and jointly regulate the supply of potassium to plants. Rapidly available potassium is easily absorbed by plants, and its abundance reflects the immediate potassium supply level of soil to plants, while slowly available potassium is the main reserve warehouse of available potassium (Römheld and Kirkby, 2010; Sandaña et al., 2020). Slowly available potassium is mainly potassium fixed by secondary minerals; it is relatively stable in the soil, but when the amount of soil available potassium is decreased by absorption and leaching, slowly available potassium is gradually released (Jin, 1993; Sparks, 2000). In recent years, with the excessive application of chemical fertilizers and the increase of replanting index in agricultural production, potassium deficiency has gradually appeared in the northern regions of China that were originally rich in potassium (Dang et al., 2014). Potassium has gradually become one of the limiting factors for increasing crop yields and ensuring the quality of agricultural products.

In this study, we used indoor cultivation and greenhouse potting to investigate the effect of chloropicrin soil fumigation on soil potassium conversion (rapidly available potassium, available potassium, and slowly available potassium), clarifying the mechanisms and characteristics of soil potassium turnover after fumigation. Knowledge about the effects of soil fumigation on different forms of soil potassium will be of value for understanding the yield-increasing effect of fumigation and guiding scientific fertilization after fumigation.

## 2. Materials and methods

### 2.1. Experimental design and sampling

Soil samples were collected from Baise, Guangxi, China (106.62° N, 23.33° E), which had been continuously planted with tomato (HongFei 6#) for 20 years. The physicochemical indicators of the soil are shown in Table 1. Soil samples were removed from crop residue and passed through a 2-mm sieve for later use. Test soil (600 g) was treated with chloropicrin at a dose of 65 mg kg^−1^. The control group did not receive any fumigant. After sealing, the soil was incubated at 28°C in darkness for 7 days. When the fumigation was finished, fumigant gas in the soil was released by ventilation. The soil was then divided into 12 cm × 12 cm potting boxes. Four treatments were set up: chloropicrin fumigated soil planted with tomato (TCP); non-fumigated soil planted with tomato (TCK); chloropicrin fumigated soil (CP); and non-fumigated soil (CK). The planted samples were each transplanted with 3–4 leaf stage tomato seedlings. All treatments had four replicates. Normal watering was conducted during the experiment, without any additional fertilization. All samples including the planted and unplanted samples were incubated in a greenhouse with the temperature of 28°C and humidity of 80%. Samples were collected on days 7, 14, 28, 42, 56, and 70; each time, 16 pots (4 treatments × 4 replicates) were taken for sampling. Plant height, stem diameter, and other growth indicators of tomato plants were recorded regularly. Rhizosphere soil from each sample was carefully collected for potassium measurement and microbiological analysis. At the end of the experiment, tomato plants were collected and the available potassium content in the plants was determined after drying.

**Table 1:** Physical and chemical properties of the tested soil.

Source of soil	Clay (%)	Silt (%)	Sand (%)	pH (1:2.5)	Salinity (us cm^−1^)	NH_4_^+^-N (mg kg^−1^)	NO_3_^−^-N (mg kg^−1^)	Organic matter (g kg^−1^)	Available phosphorus (mg kg^−1^)	Available potassium (mg kg^−1^)	Soil type^a^
Guangxi	7.5	69.6	22.9	6.95	712	15.3	49.9	36.6	317.0	256.2	Sily loam
After fumigation^b^	9.2	67.2	23.6	6.71	821	23.4	51.1	34.5	320.1	272.9	Sily loam

a Determined according to the American Soil Texture Classification Standard.

b Guangxi soil fumigated with chloropicrin for 7 days.

### 2.2. Determination of soil potassium

The methods used for determining the three forms of potassium in soil are briefly described below (Bao, 2018):

Rapidly available potassium (RK): air-dried soil (5.00 g) that had passed through a 1-mm sieve was weighed into a 100 mL-triangular flask, 50 mL of 1 mol L^−1^ NH_4_OAc solution (pH 7.0) was added, and then the flask was sealed with a rubber stopper and shaken for 30 min. The potassium content was determined using a flame photometer with a series of potassium standard solutions.

Available potassium (AK): air-dried soil (2.50 g) that had passed through a 1-mm sieve was placed in a large test-tube, 50 mL of 2 mol L^−1^ HNO_3_ was added, and then the tube was sealed with a rubber stopper and shaken for 30 min. The mixture was immediately filtered through quantitative filter paper. The filtrate was collected and the potassium content was determined using a flame photometer.

Slowly available potassium (SK): air-dried soil (2.50 g) that had passed through a 1-mm sieve was placed in a 100-mL test tube, 25 mL of 1 mol L^−1^ HNO_3_ was added, and then the tube was heated in an oil bath until it had boiled for 10 min (accurately timed from the beginning of boiling). The tube was removed from the oil bath and allowed to cool slightly. While still hot, the solution was filtered into a 100-mL volumetric flask. The soil and the test tube were washed 4–5 times with 0.1 mol L^−1^ HNO_3_ solution using 15 mL each time; this liquid was added to the volumetric flask. The volume in the flask was made up to 50 mL, and the potassium content of the solution was determined with a flame photometer.

### 2.3. Growth index and microbial community determination

During the experimental period, soil nutrient indicators, including mineral nitrogen, available phosphorus, conductivity, and others, were monitored by previously described methods (Bao, 2018).

For the root-knot nematode disease severity survey method, the tomato root system was carefully dug out of the soil and its root knot index was investigated. Knotting is divided into five levels (0–4) based on the severity of root knot occurrence and the proportion of the entire root system (Mao et al., 2016): 0 = 0%, which means that the root system is intact and without root knots; 1 = 1 to 25%, which means that there are a small number of root knots (<25% of the root system); 2 = 26 to 50%, which means that a moderate number of root knots are formed (26–50% of the root system); 3 = 51 to 75%, which means that there are a large number of root knots (51–75% of the root system); and 4 = 76 to 100%, which means that there are many and large root knots (76–100% of the root system). The formula for the root knot index is as follows: Root knot disease level (%) = 100 × Σ (the number of plants in each level × the corresponding level value) / (the total number of plants investigated × 4).

The total genomic DNA present in a 0.25-g soil sample was extracted using a MoBio Powersoil® DNA Isolation Kit (MoBio Laboratories, United States). After the DNA quality and concentration were verified by gel electrophoresis (1% agarose) and by using a NanoDrop™ 1,000 spectrophotometer (Thermo Fisher Scientific Inc., United States), genomic DNA was sequenced by using a MiSeq™ PE 250 platform at Majorbio Bio-PharmTechnology Co. Ltd. (Shanghai, China). MiSeq sequencing of 16S rRNA and internal transcribed spacer (ITS) genes was conducted using the universal primers 338F–806R and ITS1F–ITS2, respectively (Caporaso et al., 2011, 2012; Parada et al., 2016; Minich et al., 2018). The quality control and annotation of raw sequencing data were conducted using QIIME (v1.9.1; http://qiime.Org/). Briefly, reads containing ambiguous bases, those <150 bp, and those with 5′– primer mismatch, >8 contiguous matching bases, or chimera sequences, were removed. After quality control, high-quality sequences were clustered into operational taxonomic units (OTUs) with a 97% sequence similarity cutoff using UCLUST. Taxonomic information was obtained by aligning the representative sequence of each OTU with the sequences in the Silva database (Release 138.1, 2020, http://www.arb-silva.de) and UNITE (Release 8.0, 2020, https://unite.ut.ee; Chen et al., 2022). The alpha diversity index of the bacterial community in each sample was calculated after randomly resampling at 90% of the minimum sequencing depth across all samples. The raw reads were deposited in the National Center for Biotechnology Information (NCBI) Short Read Archive Database (SRP140416).

### 2.4. Data analysis

Differences in the effect of soil fumigation on soil potassium and the bacterial alpha diversity index were analyzed by analysis of variance with Tukey’s test and the SPSS statistical software package (v26.0, IBM, United States). All concentration values from the control at each time point of sampling were individually compared with the fumigation soil treatment. The indices Chao1, Ace, Shannon, and Simpson were calculated using Mothur to determine the diversity of bacterial and fungal communities. The correlation between the content of potassium and microbiological indexes as well as the tomato growth index were calculated by Spearman’s rank correlation coefficient using the package ggpubr. Co-occurrence networks were constructed based on correlation of the relative abundance of OTUs. SPARCC’s correlation coefficient (*r* ≥ 0.8 or *r* ≤ −0.8 with *p* ≤ 0.01) was used to determine pairwise associations of bacterial OTUs. In addition, various network topological indices reflecting the potential effects of chloropicrin soil fumigation on the topology of the networks were calculated in the igraph package. Visualization of the network was obtained using Gephi (v9.1). Structural equation model (SEM) was used to quantify effects of available potassium and bacterial community or fungal community on tomato growth in chloropicrin fumigated soil and non-fumigated soil. The pairwise correlation among these variables was calculated by the Mantel test using the “Ecodist” package in R platform, and a covariance matrix of these variables was inserted into AMOS 17.0 (SPSS, Chicago, IL, United States) for SEM construction and analysis.

## 3. Results

### 3.1. Changes in soil potassium conversion post-fumigation

**Figure 1:**
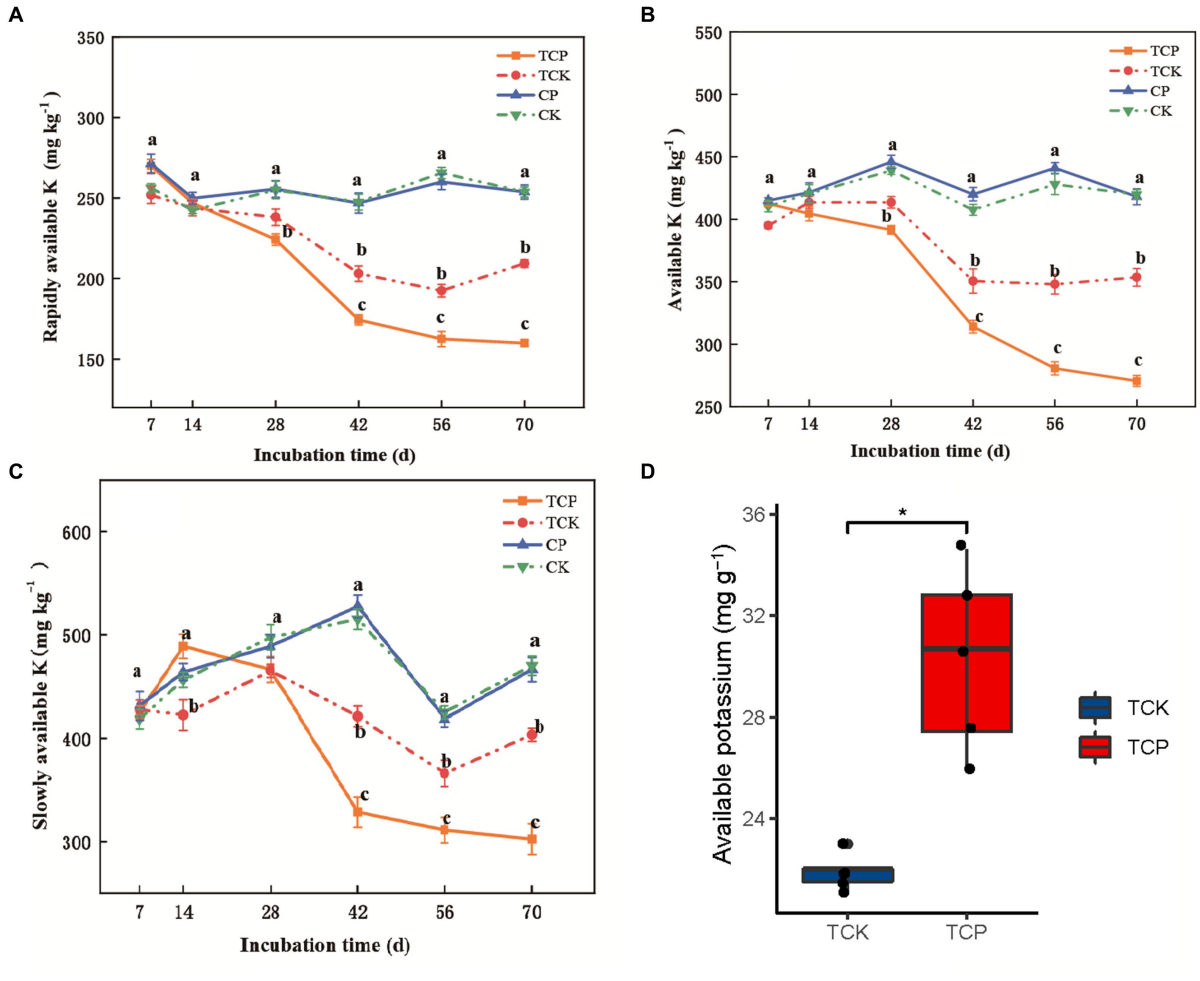
Changes over time of potassium levels in soil or tomato plants. Rapidly available potassium **(A)**, available potassium **(B)**, and slowly available potassium **(C)** in soil; available potassium **(D)** in tomato. CP: chloropicrin fumigated soil; CK: non-fumigated soil; TCP: chloropicrin-fumigated soil planted with tomatoes; TCK: non-fumigated soil planted with tomatoes. The error bars in the figure are the standard error from four replicates. *p < 0.05, **p < 0.01, ***p < 0.001, NS, no significant difference.

No significant difference in the content of rapidly available potassium, available potassium, or slowly available potassium was found between the fumigated soil and the non-fumigated soil within 70 days (Figures 1A–C). However, after planting tomato, the contents of rapidly available potassium, available potassium, and slowly available potassium in the soil of the fumigated group began to decrease on day 42, and were 14.0–24.0% (*p* < 0.001), 10.0–23.0% (*p* < 0.001), and 15.0–25.0% (*p* < 0.001) lower than those in the non-fumigated group, respectively (Figures 1A–C). We analyzed the potassium content in tomato plants on day 70, which showed that the potassium absorption by tomato was significantly higher in the fumigated soil than in the non-fumigated soil, 30.3 vs. 21.9 mg g^−1^ dry weight (*p* < 0.05; Figure 1D). This result indicates that chloropicrin fumigation significantly promotes the absorption of available potassium in the later stage of the experimental period (days 42–70 post-fumigation) by promoting the release of slowly available potassium to rapidly available potassium in the soil.

### 3.2. Change in microbial community post-fumigation

Fumigation with chloropicrin significantly reduced soil bacterial diversity (Supplementary Figure S1). After fumigation, the bacterial richness indices Chao1 and ACE, and the diversity indices Shannon and Simpson, were consistently lower than those in the non-fumigated soil from days 7 to 70 (*p* < 0.05). Moreover, planting tomatoes after fumigation resulted in further decreases in bacterial diversity (from days 28 to 70) compared with fumigation treatment without tomato planting (*p* < 0.05). The results of fungal community richness and diversity analyses showed that regardless of whether or not tomatoes were planted, the Chao1 and ACE indices were significantly lower in fumigated soil than in non-fumigated soil (Supplementary Figure S2). The Shannon and Simpson indices were significantly higher in fumigated group than in the non-fumigated group at 14–28 days, but there was no significant difference at other time points.

**Figure 2:**
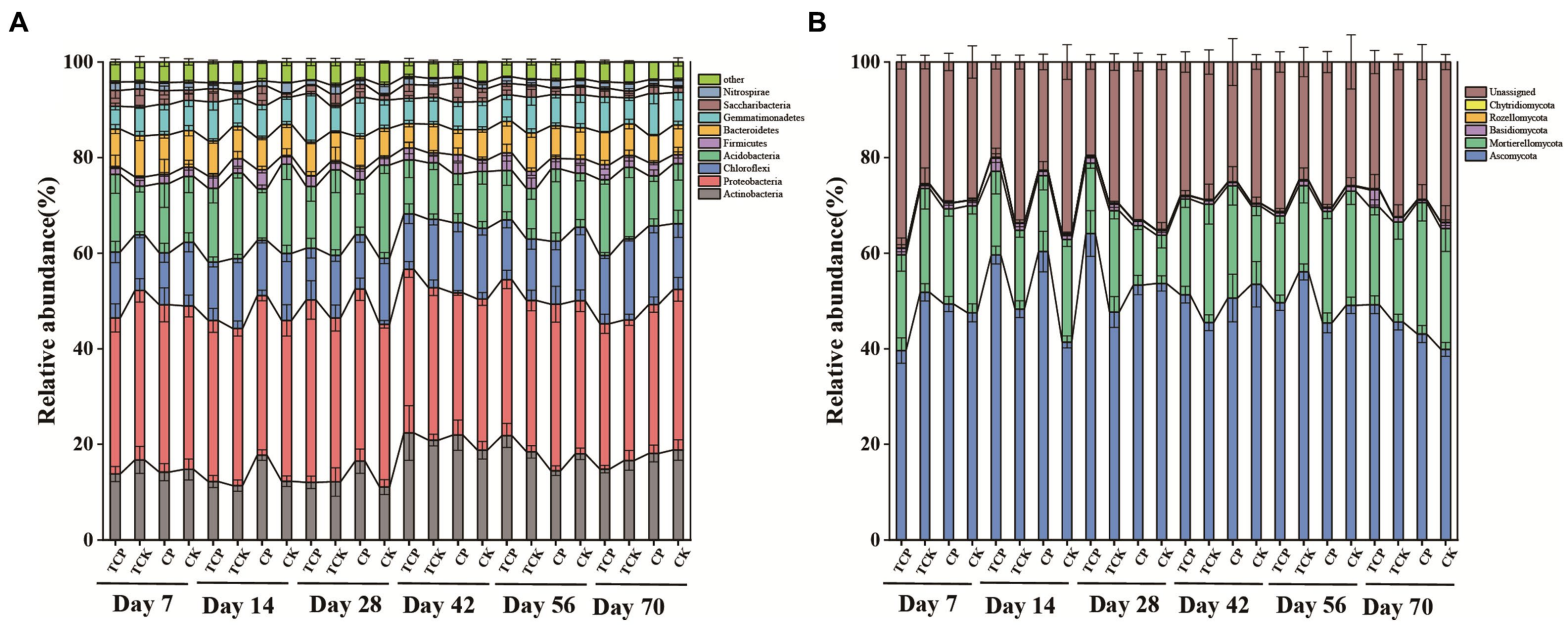
Time series of changes in microbial communities at the phylum level. **(A)** Change in bacterial relative abundance in various treatments over time. **(B)** Change in fungal relative abundance in various treatments over time. CP: chloropicrin fumigated soil; CK: non-fumigated soil; TCP: chloropicrin-fumigated soil planted with tomatoes; TCK: non-fumigated soil planted with tomatoes.

Chloropicrin significantly increased the abundance of Firmicutes and Gemmatimonadetes, by 45.4–136.2% and 19.2–36.6% respectively, compared with the non-fumigated soil (days 14–70). The stimulation effects on Firmicutes and Gemmatimonadetes were 4.8–13.9% and 33.8–42.1%, respectively, when the soil was planted with tomato (Figure 2A). The abundance of Actinobacteria increased by 16.9–49.0% on days 14–56 in chloropicrin-fumigated soil compared with non-fumigated soil, but there was no significant difference between the fumigated and non-fumigated groups when the soil was planted with tomato. The abundances of Acidobacteria and Nitrospirae decreased by 13.9–42.1% and 46.8–51.2% in chloropicrin-fumigated soil compared with non-fumigated soil, while these values changed to 4.8–27.9% and 30.8–43.1% when the soil was planted with tomato.

Considering fungi, the dominant phylum Ascomycota increased in abundance by 45.8% in chloropicrin-fumigated soil compared with non-fumigated soil at day 14, while planting tomato after fumigation reduced this increase to 23.5% (Figure 2B). Planting tomato after fumigation significantly increased the abundance of Basidiomycota, by 45.8–81.7%, compared with fumigated soil without tomato planting.

**Figure 3:**
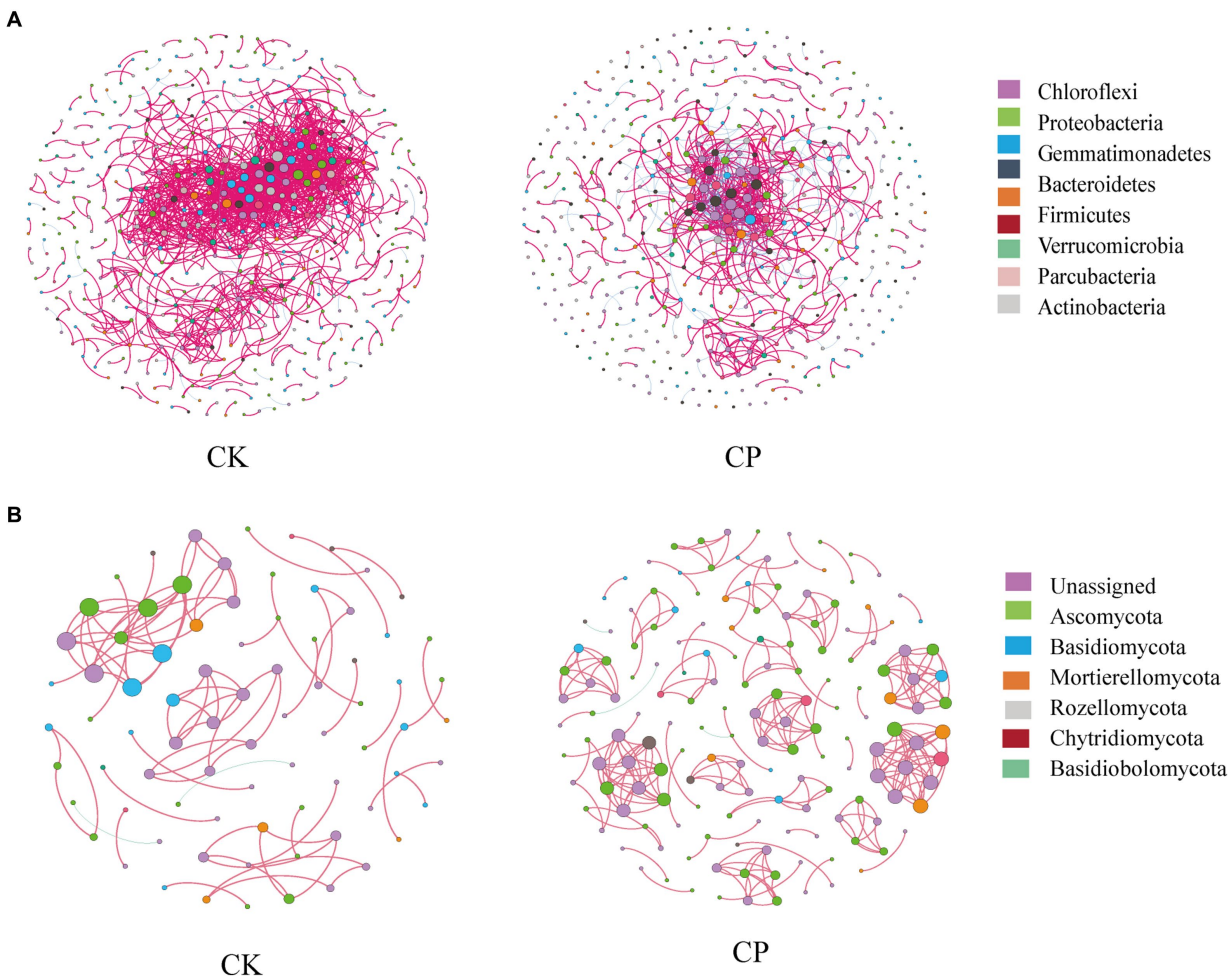
Soil microbial networks for bacteria **(A)** and fungi **(B)** in soil treated with or without chloropicrin fumigation. CK: Soil without fumigation treatment, CP: chloropicrin-fumigated soil. Phyla with relative abundance ≥5% are shown in different colors, and others are shown in gray. Details of network topological attributes are listed in Table 2.

Microbial co-occurrence network analysis showed that total links, nodes and average degree relationships between bacterial phyla were lower in fumigated than in non-fumigated soil (Figure 3A). This was evidenced by an increase in percentage negative edges and a decrease in positive edges following fumigant treatment (Table 2). In contrast, the total links, nodes and average degree relationships between fungal phyla were higher in fumigated than in non-fumigated soil (Figure 3B).

**Table 2:** Network analysis parameters in different treatments.

Taxa	Treatment	Total links	Total nodes	Positive edges (%)	Negative edges (%)	Average degree	Average weighted degree	Modularity	Average clustering coefficient
Bacterial	CK	547	1707	97.77	2.23	6.24	11.21	0.58	0.42
	CP	511	883	70.89	29.11	3.46	2.72	1.39	0.41
Fungal	CK	85	79	97.65	2.35	2.15	4.10	0.91	0.97
	CP	273	161	98.90	1.10	3.39	6.63	0.93	0.97

CP, soil fumigated with chloropicrin; CK, soil without fumigation.

**Figure 4:**
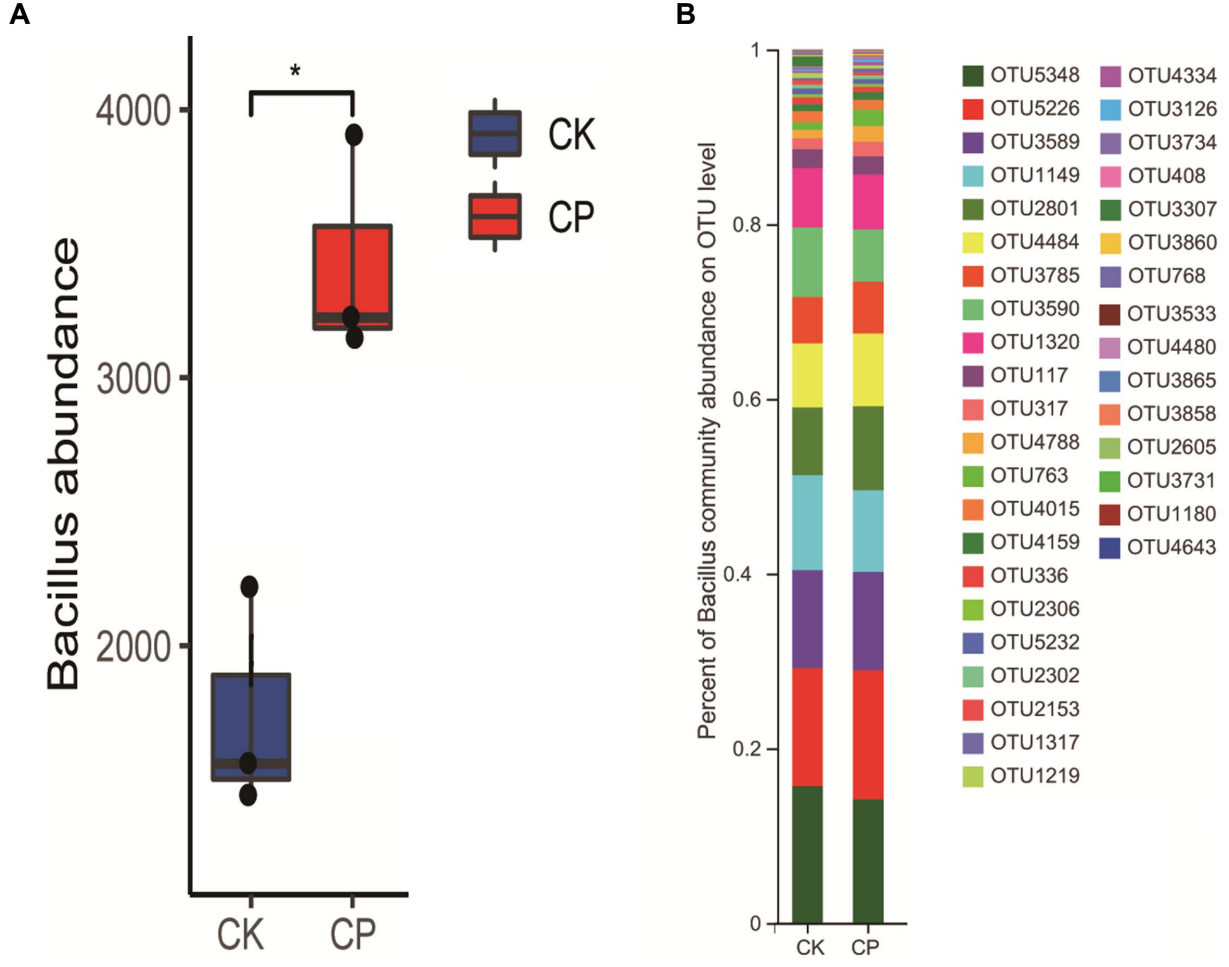
Changes in the abundance of *Bacillus* in fumigated and non-fumigated soil. **(A)** Change in *Bacillus* abundance; **(B)** change in operational taxonomic units associated with *Bacillus*. CK: control soil without fumigation treatment, CP: chloropicrin-fumigated soil. *p < 0.05, **p < 0.01, ***p < 0.001. NS indicates that there was no significant different between the control group and the fumigation group.

We also analyzed the changes in the potassium-solubilizing bacterial taxon *Bacillus* in soil with or without fumigation treatment (Figure 4). The results showed that chloropicrin soil fumigation significantly increased the abundance of *Bacillus*. For example, the species OTU1149 belong to *Bacillus* in chloropicrin fumigated soil decreased while the abundances of OTU2801 and OTU4484 significantly increased (Figure 4B).

### 3.3. Changes in soil nutrient indices post-fumigation

During the early stage post-chloropicrin fumigation (0–28 days), the ammonium nitrogen content in the fumigated soil was 2.9–5.8 times that in the non-fumigated soil. However, the ammonium nitrogen content gradually decreased during days 28–70, and recovered to the control level (5.3–7.6 mg kg^−1^, *p* = 0.407) at the end of the test period (Supplementary Figure S3A). Although the ammonium nitrogen content in fumigated soil planted with tomato also decreased gradually, the rate of decrease was slower than that in the soil without tomatoes. At the end of the test period (70 days), the ammonium nitrogen content was still significantly higher in the fumigated treatment planted with tomatoes than in the non-fumigated treatment planted with tomatoes (11.35 vs. 3.78 mg kg^−1^, *p* < 0.001; Supplementary Figure S3A).

Chloropicrin fumigation had little effect on nitrate nitrogen in the early stage post-fumigation, but the nitrate nitrogen content gradually increased during days 14–70, when it was 12.9–24.5% higher than that in the non-fumigated treatment (Supplementary Figure S3B). However, after planting tomatoes, nitrate nitrogen in the fumigated soil was 34.6–40.6% lower than it was in the fumigated treatment without tomatoes.

Chloropicrin fumigation significantly increased the available phosphorus content in the soil. For example, the available phosphorus content in the fumigated treatment was 2.0–8.0% higher than that in the non-fumigated treatment during post-fumigation period (days 14–70) (*p* < 0.05; *p* = 0.008 at day 70) (Supplementary Figure S3C). After planting tomatoes, the available phosphorus content did not differ significantly between the fumigated and non-fumigated treatments at sampling points other than day 7.

Chloropicrin fumigation significantly increased the soil electrical conductivity (EC), by 11.0–24.0% compared with that in the non-fumigated treatment, during the test period (days 7–70) (*p* < 0.001) (Supplementary Figure S3D). The soil electrical conductivity in the fumigated treatment was still significantly higher than that in the non-fumigated treatment at the end of the test period (day 70, 11.0%, *p* < 0.001). After planting tomatoes, the soil electrical conductivity in the fumigated treatment was 7.0–12.0% higher (*p* < 0.05) than that in the non-fumigated treatment at days 7–28; however, there was no significant difference in electrical conductivity between the fumigated and non-fumigated groups at days 42–70 (Supplementary Figure S3D).

### 3.4. Tomato growth and root knot index

Chloropicrin fumigation significantly promoted the growth of tomato plants (Supplementary Figure S4). After fumigation, the tomato plants were 6.3–19.4% higher than those in the control group (non-fumigated soil). The stem diameter was significantly higher than that in the control group, with an increase of 13.1–29.0% (*p* < 0.05). This ultimately led to a significant increase in tomato biomass, with the dry weight of the plants being 41.3–85.2% higher than that in the non-fumigated-soil group (*p* < 0.05).

The tested soil was severely infected with pathogens, particularly southern root-knot nematodes (*M. incognita*). In the non-fumigated group, the root-knot nematodes were discovered to be harmful at day 14, and the root-knot index was as high as 50%. The root-knot nematode disease continued to worsen over days 14–56, and the root-knot index reached 100% at the end of the experiment (day 70; Supplementary Figure S4). Chloropicrin fumigation significantly delayed the onset of root-knot nematode occurrence, pushing the onset time from day 14 to day 56. At day 70, the root-knot index in the fumigated group was 68.8%.

### 3.5. Factors driving soil potassium conversion following chloropicrin fumigation

**Figure 5:**
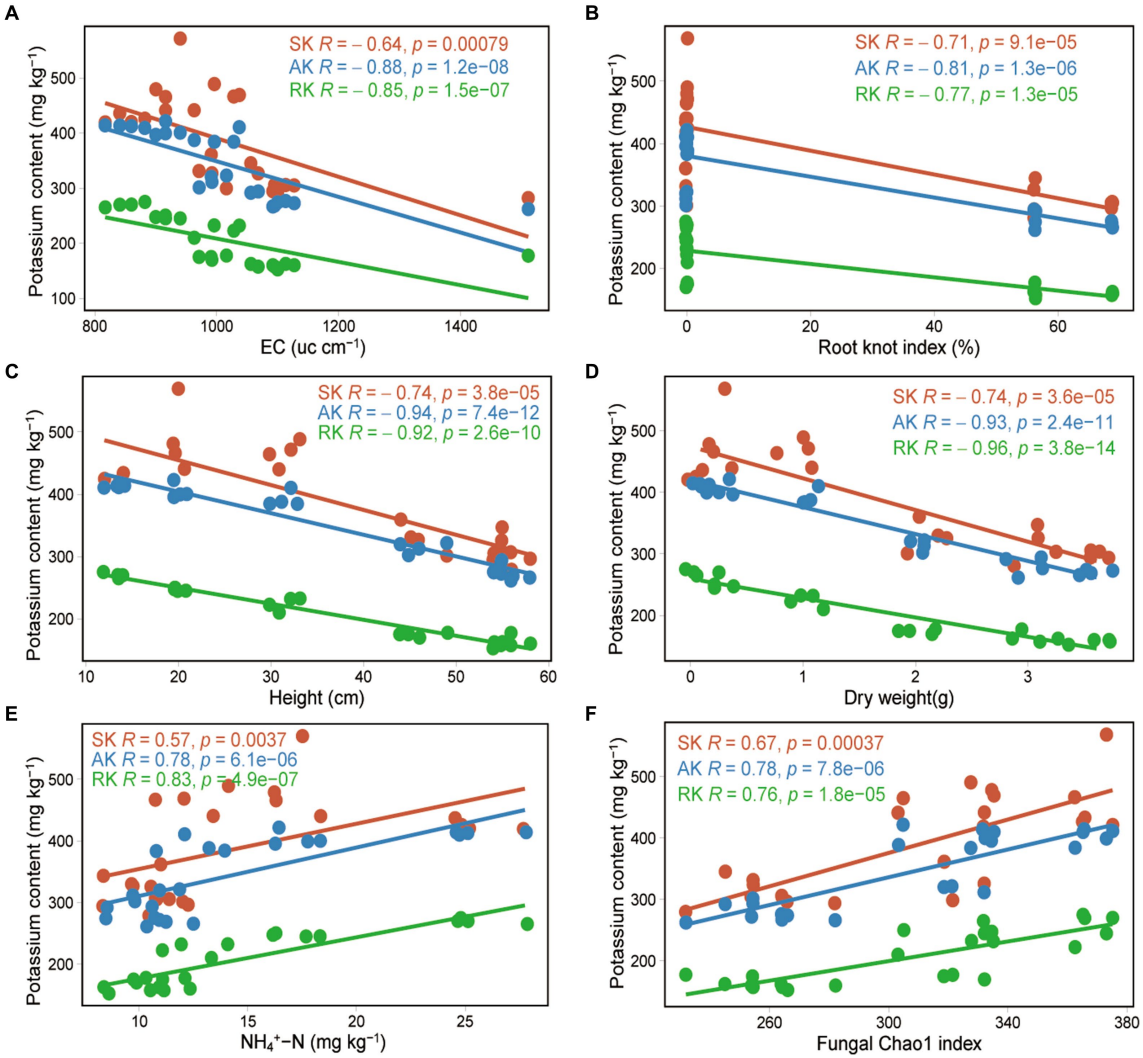
Factors driving soil potassium conversion following chloropicrin fumigation of soil. Correlation between three kinds of soil potassium and Electrical conductivity (EC) **(A)**, tomato plant root knot index **(B)**, tomato plant height **(C)**, tomato plant dry weight **(D)**, soil ammonium nitrogen **(E)**, and fungal Chao1 index **(F)**. RK, rapidly available potassium; AK, available potassium; SK, slowly available potassium.

**Figure 6:**
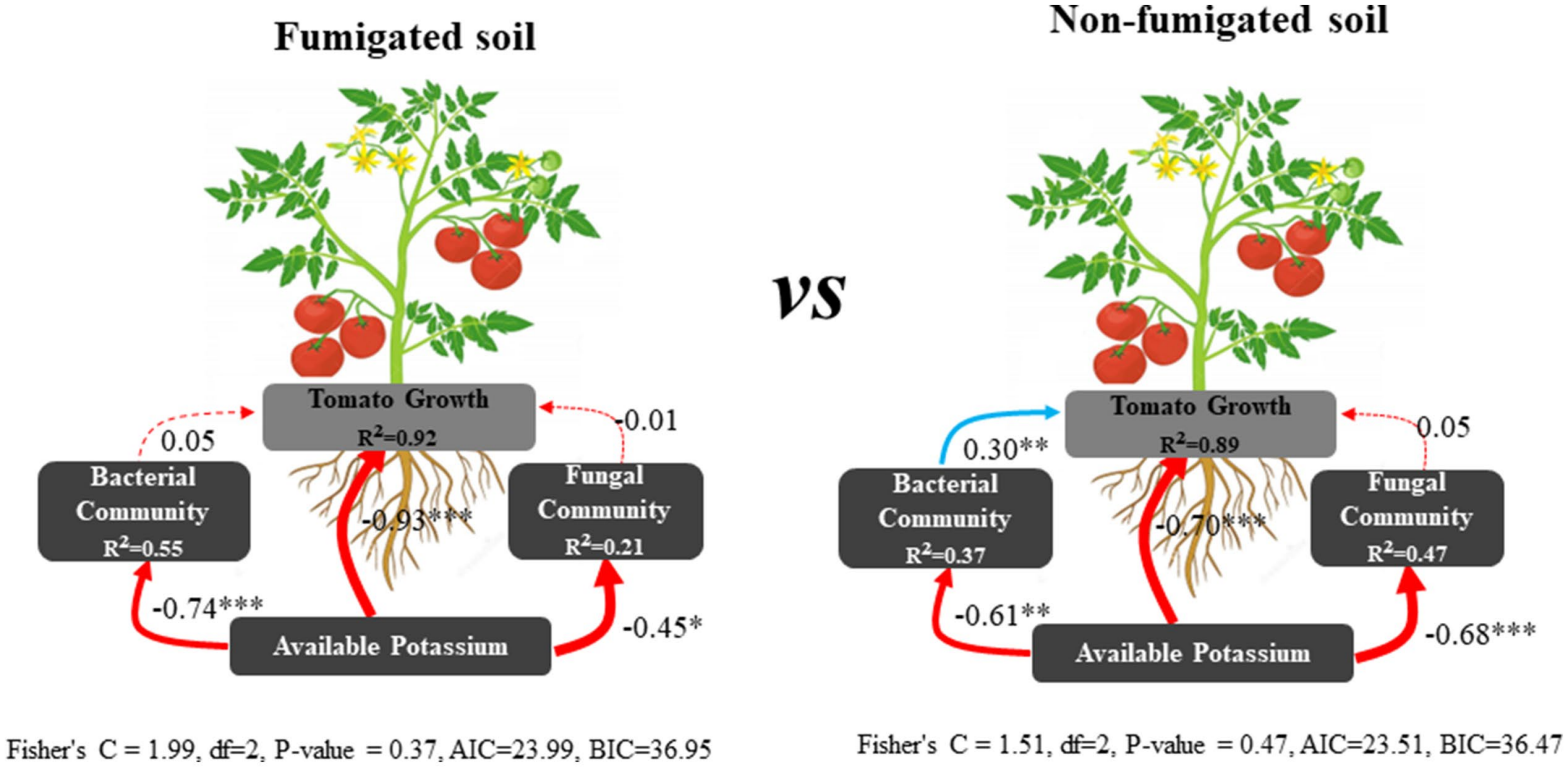
Structural equation model (SEM) illustrating the direct and indirect effects of soil potassium on tomato growth. Continuous and dashed arrows represent the significant and nonsignificant relationships, respectively. Adjacent number that are labeled in the same direction as the arrow represents path coefficients, the red and bule arrows indicate negative and positive relationships, respectively. R^2^ values indicate the proportion of variance explained by each variable. Significance levels are denoted with *p < 0.05, **p < 0.01, ***p < 0.001. Standardized total effects calculated by the SEM are displayed below the SEM.

Correlation analysis showed that soil potassium (including rapidly available potassium, available potassium, and slowly available potassium) was significantly negatively correlated with tomato plant height (correlation coefficient *r* = −0.74 to −0.94, *p* < 0.001), stem diameter (*r* = −0.68 to −0.94, *p* < 0.001), dry weight (*r* = −0.74 to −0.96, *p* < 0.001), root knot index (*r* = −0.71 to −0.81, *p* < 0.001), and EC (*r* = −0.64 to −0.88, *p* < 0.001), and significantly positively correlated with the fungal Chao1 index (*r* = 0.67 to 0.78, *p* < 0.001) and the soil ammonium nitrogen content (*r* = 0.57 to 0.83, *p* < 0.001). There was no significant correlation with bacterial diversity or soil available phosphorus content (Figure 5; Supplementary Figure S5). We observed that the content of the three forms of potassium in the soil gradually decreased as tomato plants grew, and the soil potassium content was significantly correlated with tomato growth indicators, indicating that tomato growth was an important factor driving the changes in soil potassium levels. SEM results revealed that soil potassium affect tomato growth through direct effects as well as indirect effects by influencing the bacterial community in the non-fumigated soil. However, chloropicrin fumigation enhanced the contribution of soil potassium to tomato growth and reduced the contribution of bacterial communities (Figure 6).

## 4. Discussion

### 4.1. Evidence that chloropicrin soil fumigation promotes potassium absorption

A dynamic change exists between the various forms of potassium in soil: When potassium in solution is absorbed by crops or leaches out, exchangeable potassium in the soil is released into the solution; when the concentration of rapidly available potassium decreases, the slowly available potassium in the soil is released to restore the equilibrium (Xie and Zhou, 1999). Although our experiment did not directly measure the content of insoluble potassium in the soil or the potassium content in various parts of the tomato plant, there are three lines of evidence that fumigation significantly promoted the absorption of soil potassium by tomatoes. (1) By comparing the changes in different forms of potassium content between the fumigated and non-fumigated soil groups, and between the groups with and without planted tomatoes, the content of rapidly available potassium and available potassium in the group without fumigation but with tomato planting was found to have decreased only by 14–18%, while their contents in the fumigated and tomato planted group decreased by 35–38%, indicating that fumigation promoted the consumption of soil potassium by tomatoes absorption. (2) The potassium content of tomato plants in the fumigated group was found to be significantly higher than that in the non-fumigated group (30.3 vs. 21.9 mg g^−1^ dry weight), indicating that fumigation promoted the accumulation of potassium in tomato plants. (3) Correlation analysis showed a significant negative correlation between the changes in soil potassium and growth indicators of tomato plants (plant height, stem thickness, and dry weight), indicating that tomato growth is an important factor driving changes in soil potassium. During the tomato growth process, especially in the early stage, potassium needs to be absorbed from the soil to maintain healthy growth of the crop. Previous field fumigation experiments have shown that soil fumigation can significantly increase crop yield and improve fruit quality. For example, after chloropicrin fumigation, the defect rate of tuber crops such as potatoes, ginger, and lily were significantly reduced, and the yield increased by up to 35–60% (Gullino et al., 2002; Jonathan et al., 2005; Mao et al., 2014). Increased potassium absorption by crops after fumigation may be one of the reasons for the “increased yield effect” of soil fumigation.

Potassium in soil reaches the root surface and is absorbed by plants through mass flow, diffusion, and root interception. Therefore, soil physical and chemical properties, as well as crop root biological characteristics, can affect the transport of potassium in soil, thereby affecting plant uptake of potassium. For example, soil temperature affects the diffusion of soil potassium by changing the viscosity of water, the resistance of water to potassium ion diffusion, and the average kinetic energy of particle movement (Zhan et al., 2012). Studies have shown that soil respiration is significantly enhanced after fumigation, leading to an increase in soil temperature (Li et al., 2017a,b; Zhang et al., 2019), which may promote the diffusion of potassium in soil after fumigation. In addition, the length of the main root and the number of lateral roots of tomato increased after fumigation, resulting in an increase in the contact area between roots and soil potassium, thereby enhancing uptake of potassium by the tomato plants.

### 4.2. Potential microbial mechanisms promoting potassium uptake following chloropicrin soil fumigation

The difficult-to-dissolve potassium in soil is mainly absorbed and used through the activities of microorganisms. Silicate dissolving bacteria are considered to be the main decomposers that activate difficult-to-dissolve potassium in soil (Bin et al., 2000). Silicate bacteria secrete organic acids such as oxalic acid, acetic acid, tartaric acid, and citric acid, which ionize to produce hydrogen ions or directly chelate iron, aluminum, calcium, and magnesium ions from potassium-containing minerals such as feldspar, mica, granite, and other silicates, causing them to decompose and release potassium ions, activating the difficult-to-dissolve potassium (Diep, 2013; Yao et al., 2013). Soil fumigation has a significant impact on the composition, structure, and diversity of microbial communities, which may affect the abundance and activity of microorganisms that can decompose difficult-to-dissolve potassium. For example, the abundance of *Bacillus* spores increased significantly after chloropicrin fumigation, while 1, 3-dichloropropene fumigation promoted the number of nitrogen-fixing bacteria such as *Bradyrhizobium* and *Rhizobium* (Fang et al., 2018a). *Bacillus* and *Rhizobium* have both been found to have the ability to release difficult-to-dissolve potassium. For example, *B. mucilaginosus* found in the corn rhizosphere can convert structural potassium in minerals into available potassium for absorption and use by the corn, thereby increasing its yield (Hu et al., 2013). Meanwhile, indigenous nitrogen-fixing bacteria have the ability to activate ineffective potassium in soil. Their secretion of large amounts of hydrogen ions and organic acids such as oxalic acid and malic acid can promote the dissolution of soil mineral potassium, but their activation ability varies depending on the bacterial strain (Zhang et al., 2015). Therefore, changes in the soil bacterial community structure after fumigation may affect the microbial activation and decomposition of non-exchangeable mineral potassium. Our results show that chloropicrin fumigation had a long-term inhibitory effect on microbial diversity, and the microbial abundance potentially associated with was still not recovered at the end of the test period.

Potassium-solubilizing bacteria include *B. circulans*, *B. mucilaginosus*, *Pseudomonas*, and the types of bacteria presents may vary depending on the soil type. However, *Bacillus* is the predominant genus (Gu et al., 2013). In this study, we observed that chloropicrin soil fumigation sharply changed the abundances of *Bacillus* species, including significantly decreasing the abundance of OTU1149 and increasing that of OTU2801 and OTU 4484. This change would affect the decomposition of soil slowly available potassium, thereby resulting in changes in the content of soil available potassium. However, compared with microbial indices, tomato growth indices might be more important factors driving potassium uptake. We observed that all the determined growth indexes, including dry weight, plant height, and stem diameter, were significantly negatively correlated with the soil content of potassium. Previous studies indicated that soil potassium-solubilizing bacteria promote crop growth mainly by providing nutrients for crop growth and enhancing crop stress resistance (Wu et al., 2003; Na et al., 2009). In addition, researchers have shown that the promoting effects of potassium-solubilizing bacteria on plant growth not only are reflected in an increase of available potassium content in the soil but also may be manifested in ways such as secretion of growth hormones, improvement of disease resistance, and enhancement of the rhizosphere microecological environment (Liu et al., 2006).

## 5. Conclusion

Chloropicrin soil fumigation significantly reduced the content of rapidly available potassium and available potassium in soil from around 4 weeks post-fumigation, and promoted the absorption of potassium by tomato. Chloropicrin fumigation significantly decreased soil bacterial and fungal community diversity and affected the abundances of *Bacillus* species, which are often potassium-solubilizing bacteria. Therefore, fumigation with chloropicrin enhanced the contribution of soil potassium to tomato growth and reduced the contribution of bacterial communities. Together, our research represents an important step in understanding the fertilizer effect of soil fumigation.

## Data availability statement

The datasets presented in this study can be found in online repositories. The names of the repository/repositories and accession number(s) can be found at: https://www.ncbi.nlm.nih.gov/, No. SRP124701.

## Author contributions

YS and WF designed the study and wrote the protocol. RZ, FZ, and HZ carried out determination of soil potassium. SH and JH performed the analysis of soil microbes. YS and WF managed the literature search and analyses. QW and AC analyzed the data, WF and SH were responsible for the overall design and wrote the article. All authors contributed to the article and approved the submitted version.

## Funding

This work was supported by the Pest and Disease Prevention and Control Post of Technical System of Tuber Industry in Jiangxi Province, the National Science Foundation Project of China (32001952 and 31972313), and Beijing Innovation Consortium of Agriculture Research System (BAIC01-2022-13).

## Conflict of interest

The authors declare that the research was conducted in the absence of any commercial or financial relationships that could be construed as a potential conflict of interest.

## Publisher’s note

All claims expressed in this article are solely those of the authors and do not necessarily represent those of their affiliated organizations, or those of the publisher, the editors and the reviewers. Any product that may be evaluated in this article, or claim that may be made by its manufacturer, is not guaranteed or endorsed by the publisher.
