# Supplementary figures and images for: Mechanisms by which chloropicrin fumigation promotes soil potassium conversion and absorption
Source: Front Microbiol. 2023 Jul 13;14:1208973. doi: 10.3389/fmicb.2023.1208973 (PMC10373873; doi:10.3389/fmicb.2023.1208973)

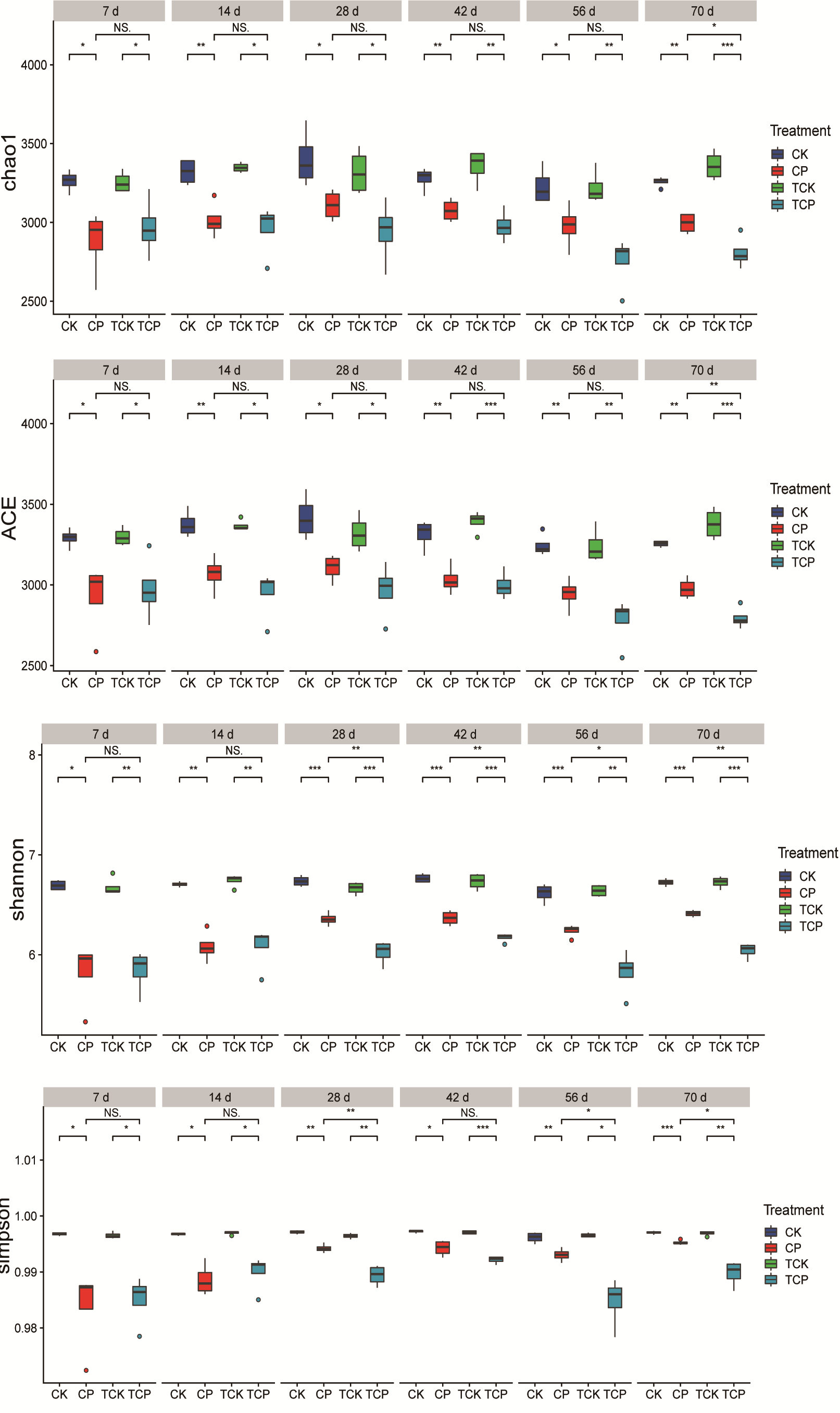

Supplement: Supplementary file 1 [file Image_1.TIF]

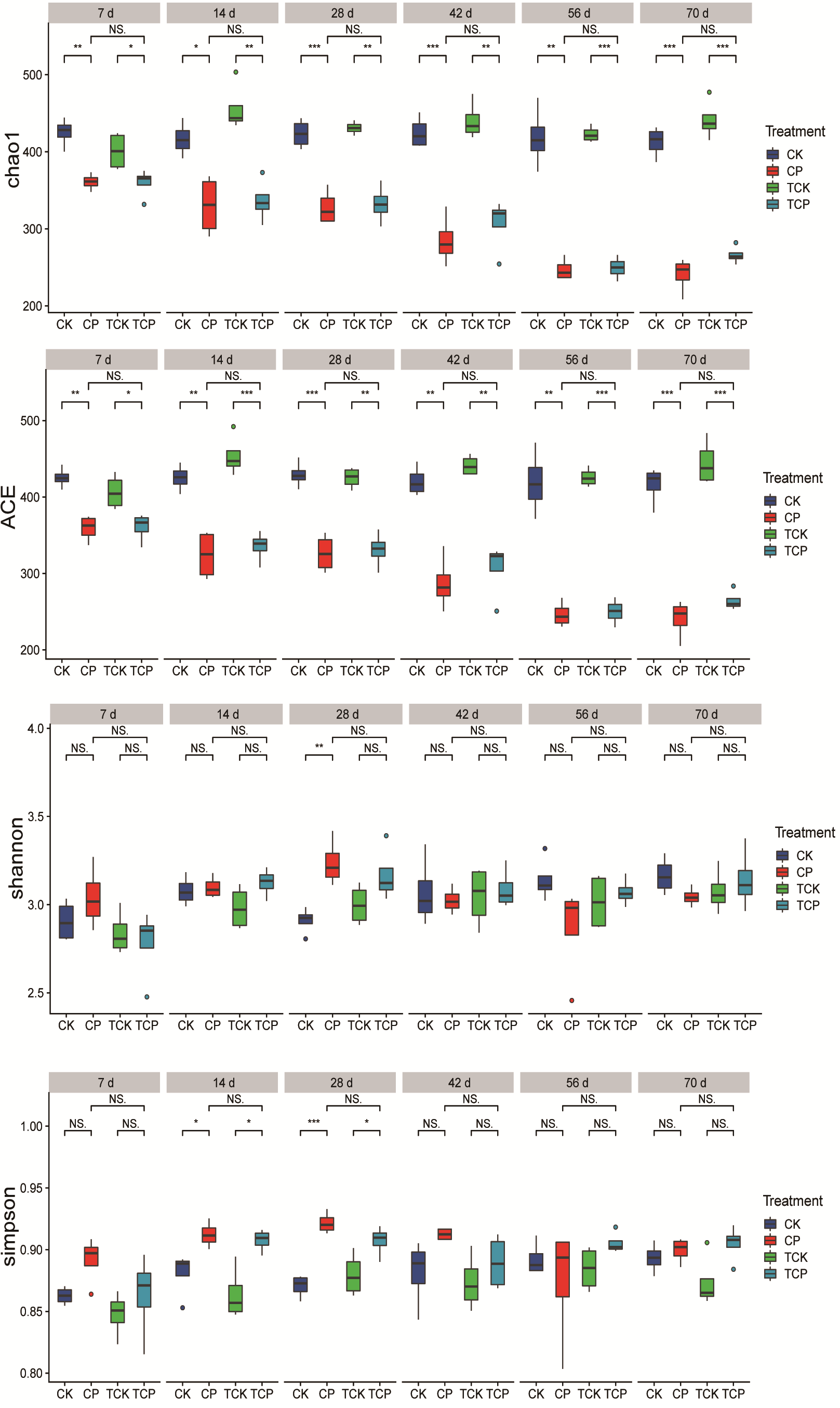

Supplement: Supplementary file 2 [file Image_2.TIF]

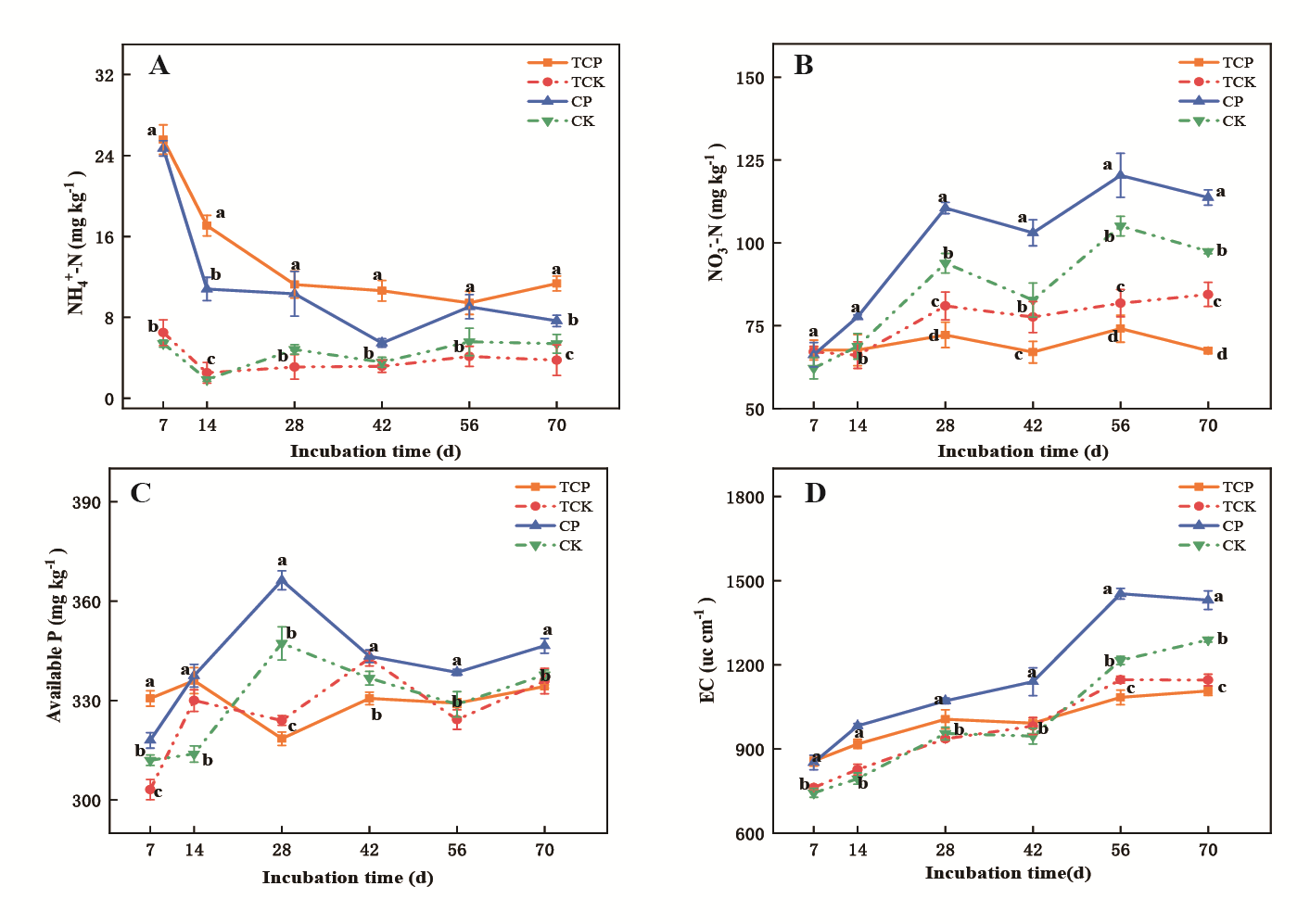

Supplement: Supplementary file 3 [file Image_3.TIF]

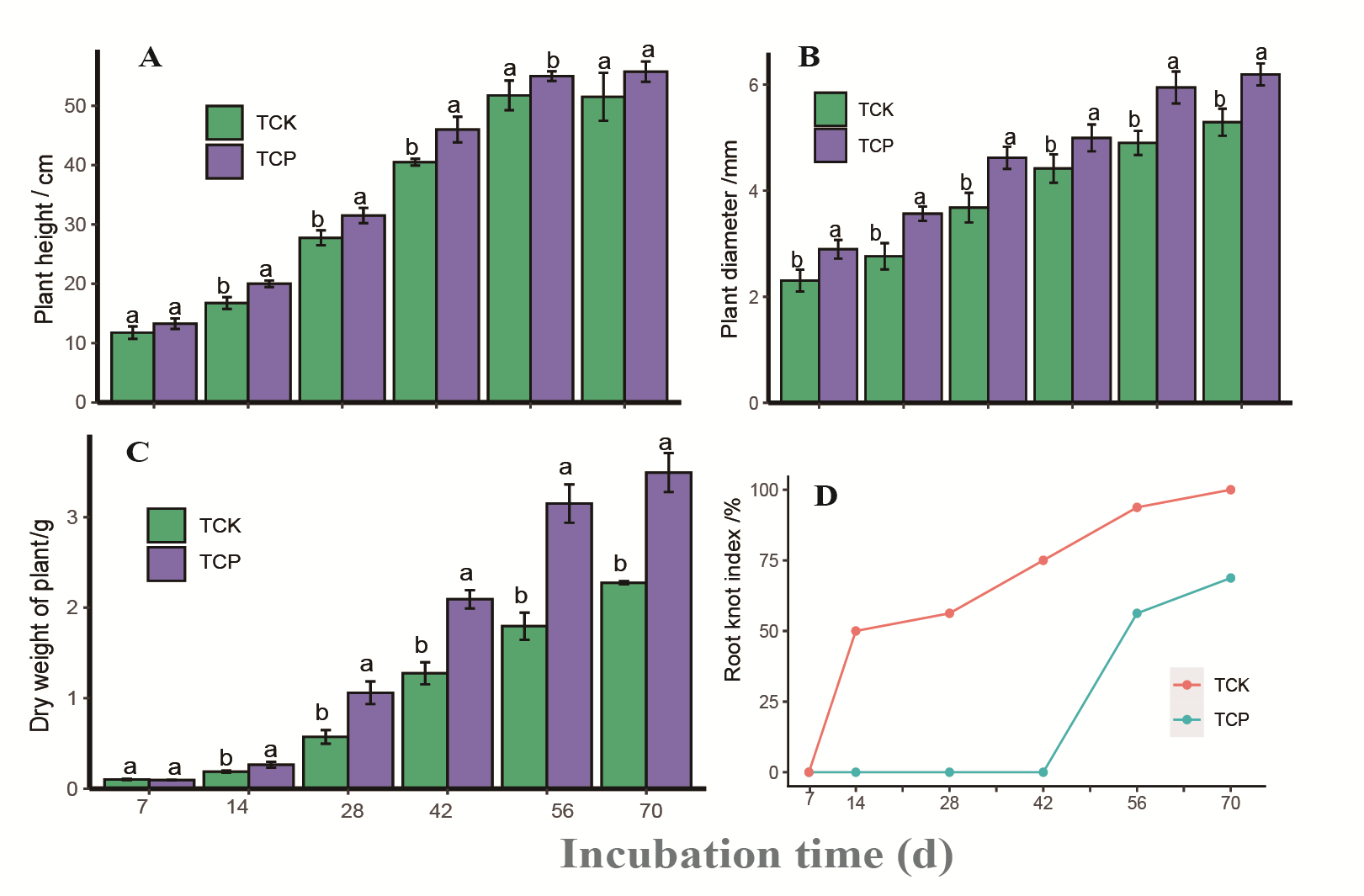

Supplement: Supplementary file 4 [file Image_4.TIF]

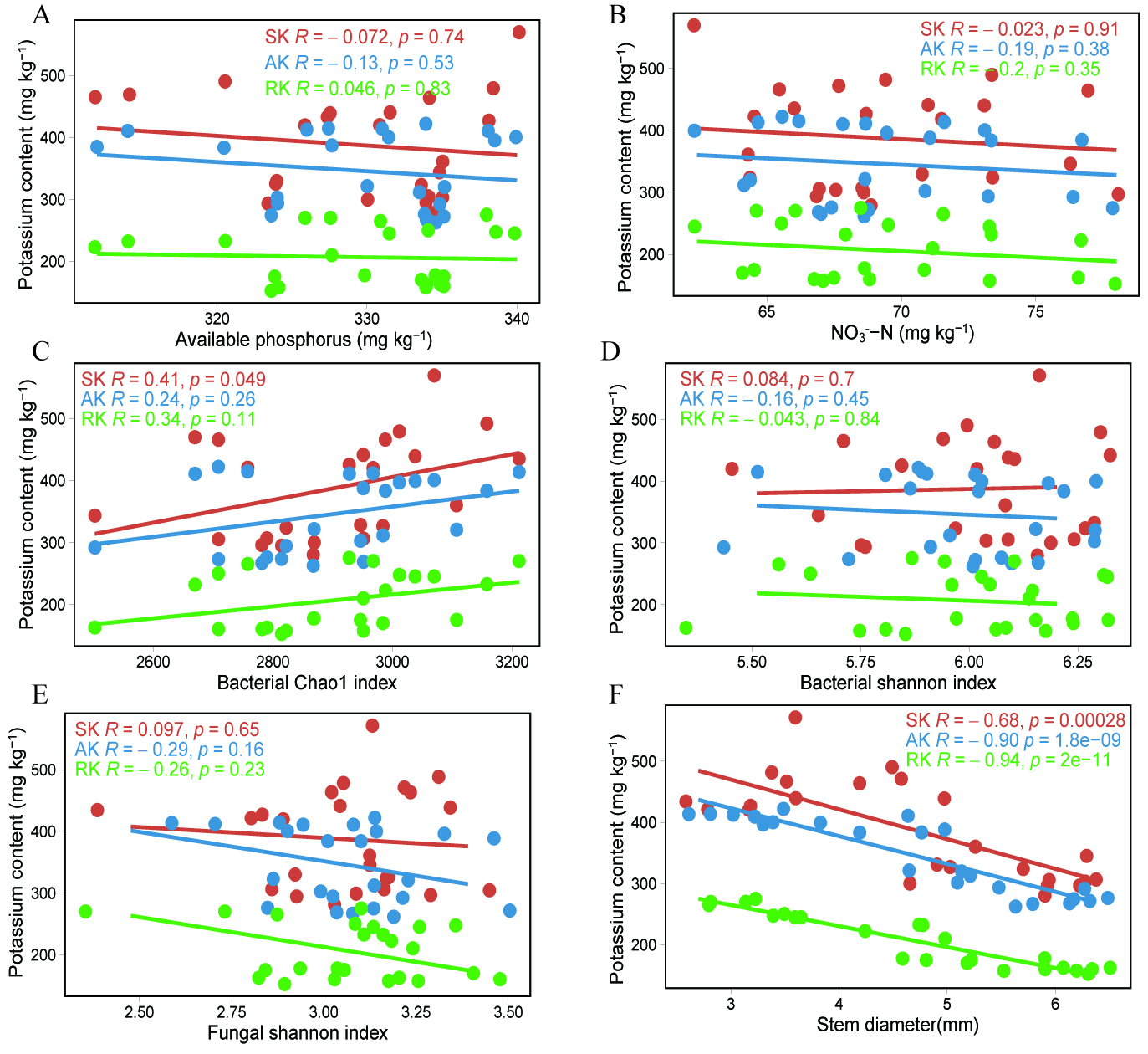

Supplement: Supplementary file 5 [file Image_5.TIF]
